# Sugammadex vs neostigmine in post-anesthesia recovery: A systematic review and meta-analysis

**DOI:** 10.17305/bb.2025.12689

**Published:** 2025-08-02

**Authors:** Ni Zhu, Yongli Li

**Affiliations:** 1 Department of Anesthesiology, Hospital of Chengdu University of Traditional Chinese Medicine, Sichuan, China

**Keywords:** Sugammadex, neostigmine, recovery, TOF, PONV, PPCs, cognitive function

## Abstract

Residual neuromuscular blockade (RNB) is linked to an increased risk of perioperative adverse events. This study systematically evaluates the impact of neuromuscular blockade antagonists on postoperative complications and quality of recovery in surgical patients. We conducted a systematic review and meta-analysis to compare the efficacy of sugammadex and neostigmine. Comprehensive searches were performed across medical databases, including Web of Science, PubMed, Embase, and the Cochrane Library, with a final search date of April 6, 2025. A total of 35 randomized controlled trials (RCTs) involving 4275 patients, along with two retrospective studies comprising 49,642 participants, met the inclusion criteria. The meta-analysis revealed that sugammadex facilitated faster reversal of RNB compared to neostigmine, as indicated by a quicker recovery to a Train-of-Four ratio (TOFR) ≥ 0.9 (standardized mean difference [SMD] −3.45; 95% confidence interval [CI], −4.42 to −2.48), a shorter extubation time (SMD −1.44; 95% CI, −2.02 to −0.85), and a decreased incidence of RNB (risk ratio [RR] 0.18; 95% CI, 0.07 to 0.47). Moreover, sugammadex significantly reduced postoperative complications compared to neostigmine, including the incidence of postoperative nausea and vomiting (PONV) (RR 0.64; 95% CI, 0.46 to 0.88), postoperative pulmonary complications (PPCs) (RR 0.62; 95% CI, 0.38 to 0.99), and bradycardia (RR 0.32; 95% CI, 0.20 to 0.50). In conclusion, sugammadex provides a faster reversal of neuromuscular blockade compared to neostigmine and is associated with a reduction in postoperative complications. However, this expedited reversal does not result in measurable improvements in overall recovery quality, nor do either sugammadex or neostigmine significantly affect postoperative cognitive function.

## Introduction

Each year, over 230 million major surgical procedures are performed worldwide, the majority of which necessitate general anesthesia [1]. Neuromuscular blocking agents (NMBAs) play a critical role in this setting by facilitating endotracheal intubation, inducing muscle relaxation, and ensuring optimal conditions for surgery. However, the administration of NMBAs has been associated with negative postoperative outcomes, particularly residual neuromuscular blockade (RNB) [2–4]. RNB has been linked to an increased risk of pulmonary complications, higher mortality rates, extended hospital stays, elevated healthcare costs, and a greater overall medical burden [5].

Neostigmine, a cholinesterase inhibitor, is commonly utilized as a reversal agent for RNB, facilitating a more rapid recovery following general anesthesia. Despite its clinical significance, residual blockade occurs in approximately 40% of patients even after neostigmine administration [6]. This residual blockade, even when mild, can compromise respiratory function, swallowing, and the ability to maintain a patent airway, particularly in elderly patients. Such impairments considerably elevate the risk of postoperative complications, including pneumonia, aspiration, and atelectasis [7–9].

Sugammadex, introduced in 2008, is a gamma-cyclodextrin that selectively binds to rocuronium, enabling rapid and complete reversal of neuromuscular blockade without negatively impacting the function of upper airway dilators [10, 11]. When compared to neostigmine, sugammadex demonstrates superior efficacy in achieving a Train-of-Four ratio (TOFR) greater than 0.9 [12]. However, its effects on broader clinical outcomes remain unclear. While some studies indicate potential advantages of sugammadex for postoperative recovery, others present inconclusive or conflicting results [13].

Numerous meta-analyses have compared sugammadex and neostigmine concerning specific postoperative outcomes, such as pulmonary complications, postoperative nausea and vomiting (PONV), and train-of-four recovery [14–16]. However, significant gaps remain: (1) a lack of evidence in high-risk populations, including bariatric patients, individuals with high American Society of Anesthesiologists (ASA) status, and the elderly; (2) an absence of integrated assessments of multidimensional recovery; and (3) unaddressed methodological heterogeneity. To address these limitations comprehensively, we conducted the most extensive systematic review and meta-analysis to date. Our study uniquely: (i) compares the agents in underrepresented high-risk cohorts through subgroup analyses; and (ii) synthesizes evidence across critical recovery domains—such as pulmonary complications, PONV, recovery scores, cognitive function, and discharge metrics—providing a holistic view of convalescence. This approach offers tailored evidence for complex clinical decisions, where the choice of agent significantly impacts recovery, thereby advancing precision anesthesia practice beyond broad efficacy comparisons.

## Materials and methods

The study protocol has been pre-registered with the international prospective register of systematic reviews (PROSPERO) database (registration number CRD42024561006). This research design followed the guidelines set forth by the Preferred Reporting Items for Systematic Reviews and Meta-Analyses (PRISMA), ensuring rigorous and comprehensive reporting [17].

### Inclusion and exclusion criteria

This systematic review utilized the PICOS framework to establish eligibility criteria. We included studies involving patients (P) undergoing general anesthesia who required reversal of neuromuscular blockade. The interventions (I) and comparators (C) were defined as follows: for trials evaluating sugammadex, the comparator was neostigmine; for trials assessing neostigmine, the comparators were either placebo or standard care (without an active reversal agent). Eligible studies were required to report at least one primary outcome (time to TOFR ≥ 0.9 or extubation time) or secondary outcomes, which included the incidence of RNB, hospital length of stay, duration in the recovery or operating room (OR), quality of recovery scores, incidence of PONV, postoperative pulmonary complications (PPCs), bradycardia, 30-day hospital readmission, or cognitive outcomes assessed by tools such as the Mini-Mental State Examination (MMSE) or the Montreal Cognitive Assessment (MoCA). In terms of study design (S), we included randomized controlled trials (RCTs) comparing sugammadex to neostigmine or neostigmine to placebo/control. Acknowledging the potential scarcity of RCTs specifically measuring cognitive outcomes, high-quality retrospective cohort studies (as determined by the Newcastle–Ottawa Scale [NOS]) were also included solely for the analysis of cognitive outcomes. Studies were excluded if research data were unavailable or non-extractable, if they were deemed low quality by standardized assessment tools (Cochrane RoB 2.0 for RCTs; NOS score < 5 for cohort studies), or if they were non-human studies, case reports, reviews, or conference abstracts.

### Search strategy

Comprehensive literature searches were conducted across multiple databases, including Web of Science, PubMed, EMBASE, and the Cochrane Library, up until April 6, 2025. This search encompassed both published works and preprints. The complete search strategy is detailed in Supplementary material 1.

### Data extraction and quality assessment

Data extraction from the selected articles was conducted using a data collection table by one investigator and subsequently verified by a second investigator. Each study included in this review was evaluated for risk of bias by two independent investigators utilizing the Cochrane quality assessment tool, specifically designed for RCTs. The NOS was employed to assess the quality of retrospective cohort studies. The studies were classified into two categories: ‘low risk’ and ‘high risk.’ Initial disagreements were resolved through structured discussions between the reviewers, referencing pre-defined inclusion and exclusion criteria, while persistent disagreements (less than 5% of cases) were adjudicated by a senior investigator, whose decision was final.

### Statistical analysis

For continuous variables, we analyzed the available data by aggregating it to calculate the mean difference (MD) using a random effects model, accompanied by a 95% confidence interval (CI). In contrast, for dichotomous variables, we compiled the data to estimate a pooled risk ratio (RR), again utilizing a random effects model with a 95% CI. To evaluate heterogeneity among the studies, we employed the Cochran-based *I*^2^ statistic and the chi-square test; a *P* value exceeding 0.10 and an *I*^2^ statistic below 50% were interpreted as indicators of low heterogeneity. When more than 10 studies were included, we conducted Egger’s and Begg’s tests to assess potential publication bias. Statistical analyses were performed using Stata software (version 15.0; StataCorp LLC, College Station, TX, USA).

## Results

### Study selection

**Figure 1.**
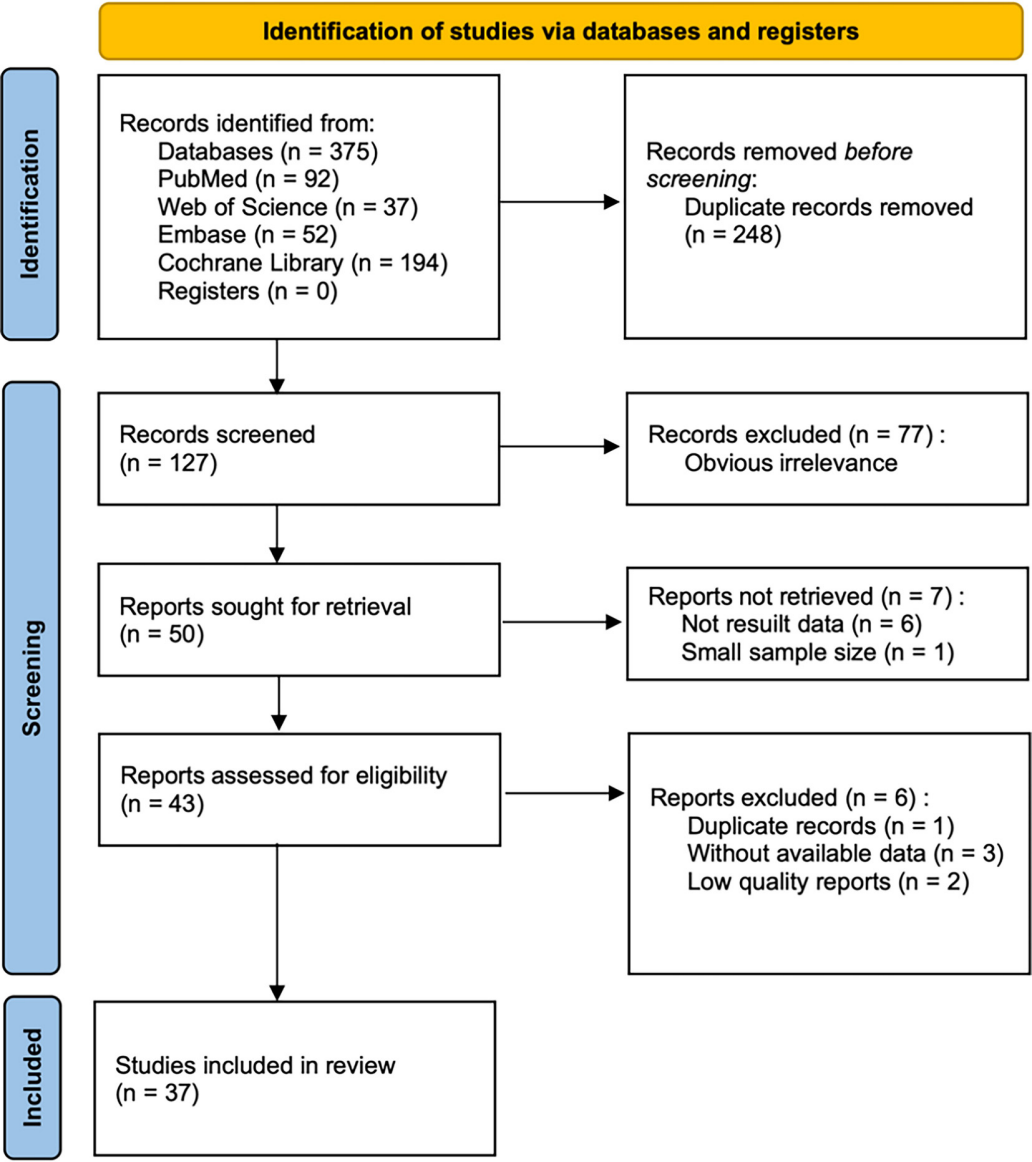
Flowchart illustrating the study selection process.

The procedure for retrieving results and selecting research articles is detailed in the flowchart shown in Figure 1. Initially, 375 potentially relevant articles were identified through the literature search. Ultimately, 37 studies were included in the review for analysis.

**Figure 2.**
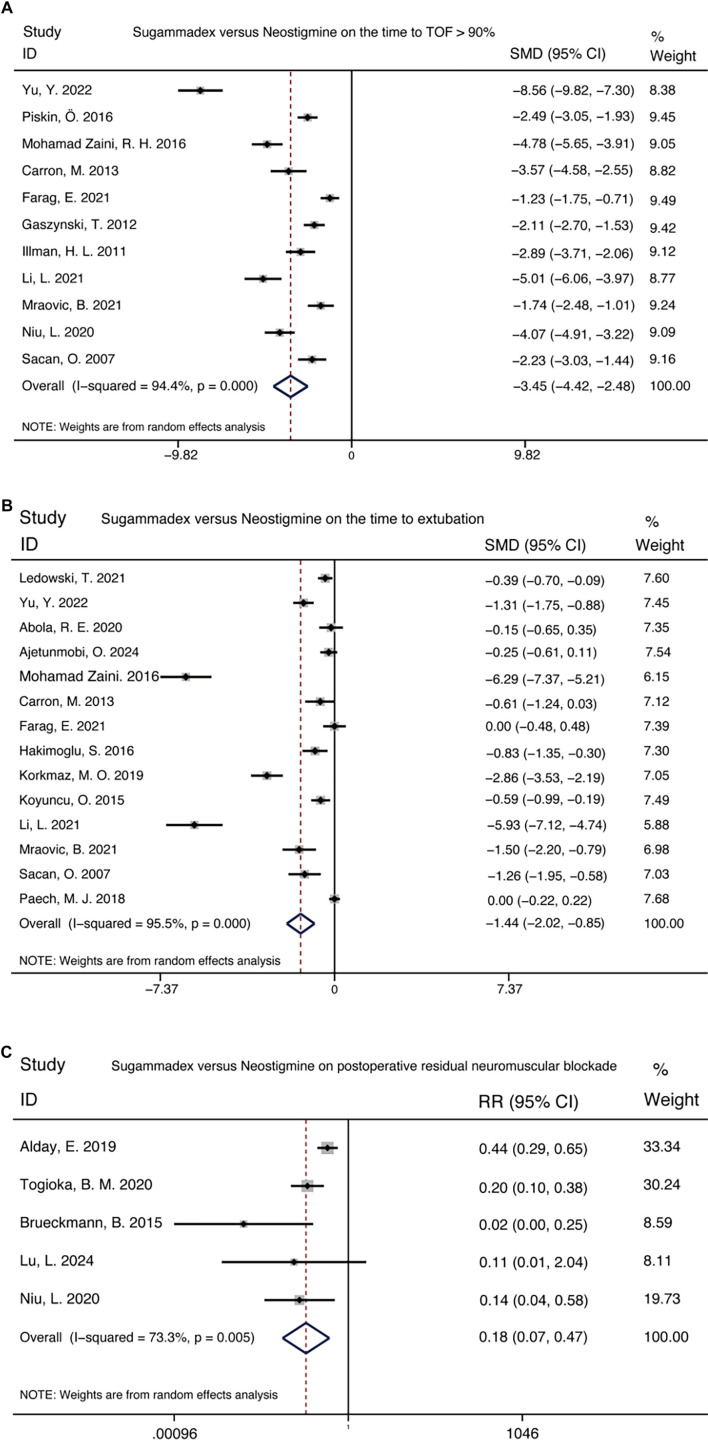
**Comparative assessment of recovery speed between sugammadex and neostigmine, evaluated through TOF ratios and extubation times.** MD with 95% CIs are shown, pooled using a random-effects model. The diamond represents the overall effect estimate. Abbreviations: TOF: Train-of-four; MD: Mean difference; CI: Confidence interval.

**Table 1:** Pooled analysis of comparative outcomes between sugammadex and neostigmine

**Outcomes**	**Cohorts**	**Participants**	**Pooled effect size**	**95% CI**	***I* ^2^**
Time of TOF ≥0.9	11	700	MD ═ −3.45	−4.42 to −2.48	94.4
Extubation time	14	1312	MD ═ −1.44	−2.02 to −0.85	95.5
PACU stay	7	565	MD ═ −0.20	−0.62 to 0.23	83.5
OR stay	3	260	MD ═ −0.60	−1.20 to 0.01	80.8
Hospital length of stay	9	991	MD ═ −0.32	−0.70 to 0.07	88.6
Recovery scores	3	443	MD ═ −0.12	−0.43 to 0.19	50.2
RNB incidence	5	608	RR = 0.18	0.07–0.47	73.3
PONV incidence	16	1986	RR = 0.64	0.46–0.88	53.6
PPCs incidence	6	802	RR = 0.62	0.38–0.99	30.3
Bradycardia incidence	6	948	RR = 0.32	0.20–0.50	0.0
30-day readmission rate	2	300	RR = 0.39	0.17–0.92	0.0
Cognitive impairment rate (S vs N)	4	49953	RR = 1.09	0.77–1.54	35.2
Cognitive impairment rate (N vs C)	3	635	RR = 0.66	0.36–1.21	54.8

Abbreviations: TOF: Train-of-Four (neuromuscular monitoring); PACU: Post-anesthesia care unit; OR: Operating room; RNB: Residual neuromuscular blockade; PONV: Postoperative nausea and vomiting; PPCs: Postoperative pulmonary complications; MD: Mean difference; RR: Risk ratio; CI: Confidence interval.

### Study characteristics

The characteristics of the included studies are summarized in Supplementary material 2. A total of 37 articles were considered for the meta-analysis, comprising 35 RCTs involving 4275 patients and two retrospective studies with 49,642 participants. Among these studies, 11 focused on the time required to achieve a TOFR of 0.9 [7, 12, 18–26], 14 examined extubation time [5, 7, 12, 13, 19, 20, 23, 24, 26–31], 5 assessed the incidence of RNB [8, 9, 25, 32, 33], 9 reported on the length of hospital stay [5, 7–9, 13, 23, 24, 34, 35], 7 investigated post-anesthesia care unit (PACU) duration [7, 13, 20, 24, 34–36], 3 analyzed OR time [13, 24, 35], and 3 evaluated quality of recovery scores [27, 31, 34]. Furthermore, 16 studies addressed the incidence of PONV [5, 8, 13, 19, 23, 26, 29, 31–33, 35–40], and 6 investigated the occurrence of PPCs [5, 7–9, 23, 32]. Additionally, only 2 studies reported the incidence of 30-day hospital readmissions [7, 8], 6 focused on the prevalence of bradycardia [23, 30, 32, 39, 41, 42], and 7 evaluated postoperative cognitive impairment [32, 43–48]. Among the 7 studies reporting cognitive outcomes, 3 assessed postoperative delirium (POD) using the confusion assessment method (CAM) or brief CAM (bCAM) daily from postoperative days 1–7, while 4 studies evaluated postoperative cognitive dysfunction (POCD) using the MMSE or MoCA, with impairment defined as postoperative Z-scores ≤ −1.96; measurements varied but commonly occurred on days 1, 3, and 7.

### Risk of bias and quality assessment

The evaluation of bias revealed a minimal risk for most studies included in this analysis. Detailed information regarding the individual studies, their outcomes, and the associated risk of bias is available in Supplementary material 3.

### Results of pooled analysis

Table 1 presents a summary of the key results. The pooled outcome analyses revealed similar recovery profiles for sugammadex and neostigmine across several parameters, including PACU duration, OR time, hospital length of stay, postoperative cognitive function, and recovery scores, all of which exhibited no statistically significant differences. However, sugammadex demonstrated significant advantages in critical recovery metrics: patients attained a TOF ratio of ≥ 0.9 more rapidly, experienced shorter extubation times, and had lower rates of postoperative complications, including PONV, PPCs, and bradycardia.

### Comparison of speed and quality of recovery

Sugammadex demonstrated a more rapid reversal of neuromuscular blockade compared to neostigmine, achieving a TOFR of ≥ 0.9 in 11 trials (standardized mean difference [SMD] −3.45 [−4.42 to −2.48]) and reducing extubation time across 14 trials (SMD −1.44 [−2.02 to −0.85]). Additionally, sugammadex significantly lowered the risk of RNB when compared to neostigmine, as evidenced by five trials showing a relative risk (RR) of 0.18 [0.07–0.47] (Figure 2). However, pooled analyses revealed that parameters such as hospital stay (9 trials, SMD −0.32 [−0.70 to 0.07]), recovery room duration (7 trials, SMD −0.20 [−0.62 to 0.23]), OR duration (3 trials, SMD −0.60 [−1.20 to 0.01]), and quality of recovery scores (3 trials, SMD −0.12 [−0.43 to 0.19]) were comparable between sugammadex and neostigmine (Figure S1).

### Comparison of the incidence of postoperative complications

**Figure 3.**
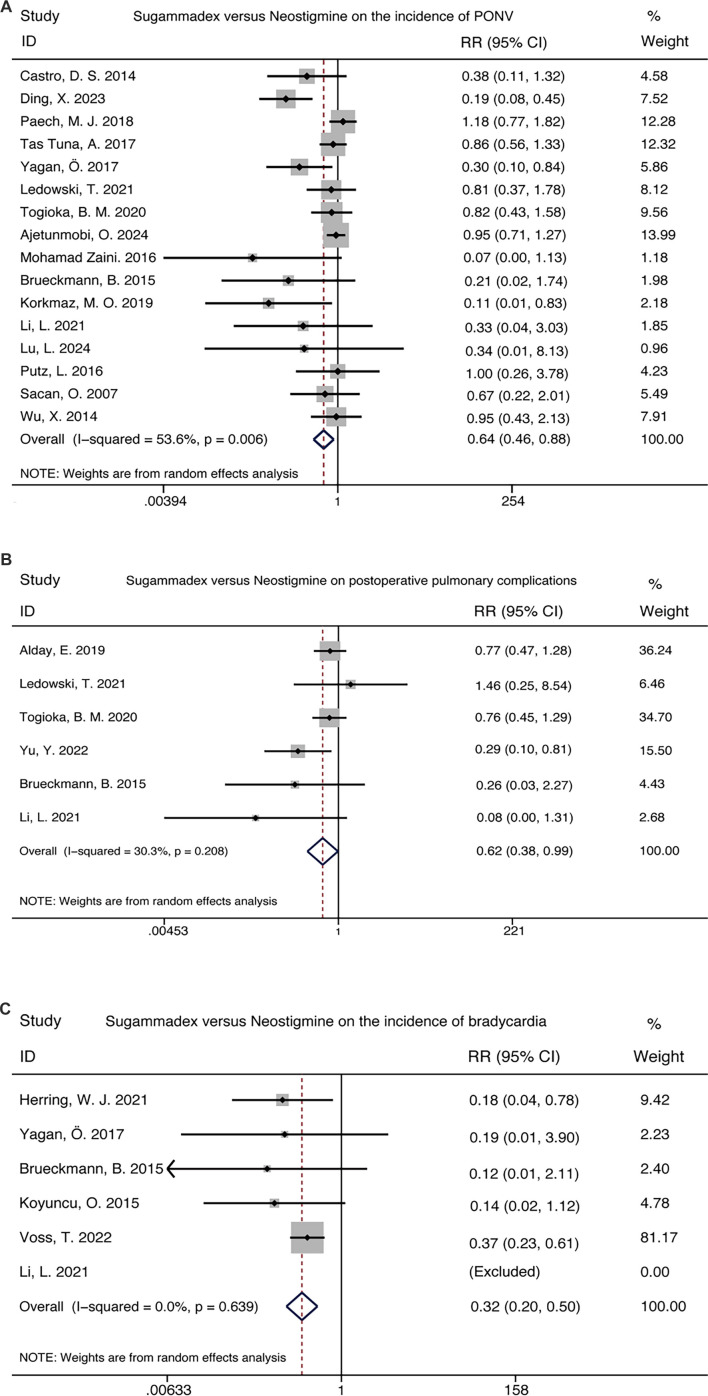
**Comparative incidence of postoperative complications between sugammadex and neostigmine.** A study reporting zero events in both arms was omitted from the analysis. RR with 95% CIs are pooled using a Mantel-Haenszel random-effects model. The diamond indicates overall effect size. Abbreviations: RR: Risk ratio; CI: Confidence interval.

Sugammadex significantly decreased the risk of postoperative complications, including the incidence of PONV (16 trials, RR 0.64 [0.46–0.88]), PPCs (6 trials, RR 0.62 [0.38–0.99]), and bradycardia (6 trials, RR 0.32 [0.20–0.50]) (Figure 3).

### Comparison of cognitive function and long-term outcomes

**Figure 4.**
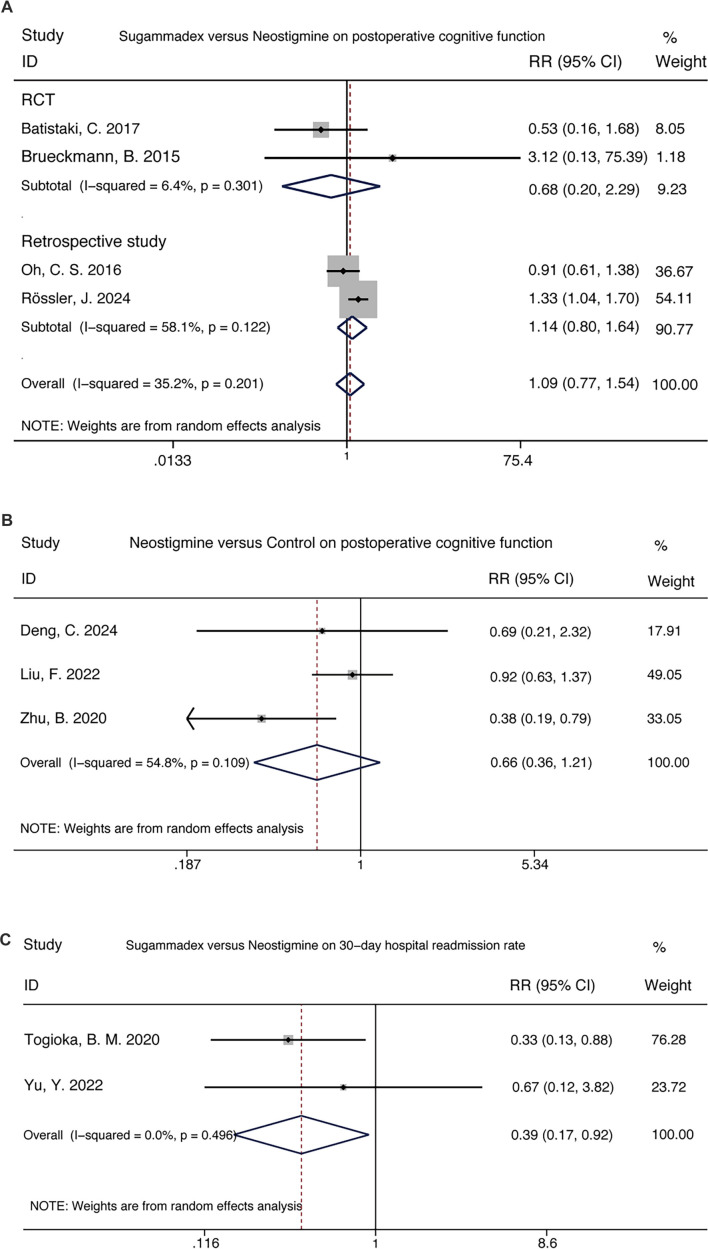
**Comparison of sugammadex and neostigmine regarding postoperative cognitive function and long-term recovery outcomes, illustrating their respective impacts on cognitive performance and extended health indicators.** RR with 95% CIs are pooled using a Mantel-Haenszel random-effects model. The diamond indicates overall effect size. Abbreviations: RR: Risk ratio; CI: Confidence interval.

A comparison of sugammadex and neostigmine regarding cognitive impairment revealed similar outcomes (4 trials, RR 1.09 [0.77–1.54]). However, sugammadex was associated with a significantly lower 30-day readmission rate compared to neostigmine (2 trials, RR 0.39 [0.17–0.92]). Additionally, neostigmine did not demonstrate improved cognitive outcomes when compared to placebo (3 trials, RR 0.66 [0.36–1.21]) (Figure 4).

### Results of the subgroup analysis

Due to the significant heterogeneity observed in our primary outcomes, specifically the time to TOF ratio ≥ 90% and extubation time, we conducted subgroup analyses stratified by age, ASA physical status classification, and body mass index (BMI). The results indicated that the findings within the subgroups aligned with the overall results: Sugammadex facilitated a more rapid attainment of a TOF ratio of 90% compared to neostigmine (Figure S2A–2C). Lower heterogeneity was noted in the subgroup of patients aged ≤ 14 years, ASA class ≥ 3, and those with a BMI of 30–40 kg/m^2^. Additionally, extubation time was significantly shorter in the sugammadex group relative to the neostigmine group (Figure S3A–3C), with reduced heterogeneity specifically observed in the subgroup with a BMI ≥ 40 kg/m^2^.

### Analysis of publication bias

The analysis performed using Egger’s and Begg’s tests indicated no significant publication bias for any of the primary outcomes, as evidenced by a *P* > 0.05.

## Discussion

We conducted a systematic review and meta-analysis to evaluate the effects of neuromuscular blockade antagonists on postoperative complications and the overall quality of patient recovery. The analysis revealed that sugammadex was more effective than neostigmine in reversing neuromuscular blockade and was associated with a reduction in postoperative complications. However, this rapid reversal did not lead to significant differences in overall recovery. Furthermore, both sugammadex and neostigmine appeared to have minimal impact on postoperative cognitive function, with neostigmine showing no substantial improvement in cognitive outcomes.

The findings of this study corroborate earlier systematic reviews [15, 16, 49], which also reported a comparable reduction in postoperative complications associated with sugammadex. However, these prior studies did not assess its impact on various factors, including the duration of hospital stays, PACU stays, patient-reported satisfaction, cognitive function, or the incidence of RNB. This study provides valuable insights by demonstrating that sugammadex is more effective than neostigmine in reversing neuromuscular blockade and is associated with a decrease in postoperative complications. Nevertheless, this increased reversal speed does not necessarily translate into differences in overall recovery efficiency [16]. Liu et al. [47] found no significant differences in mortality rates within a six-month period between the neostigmine and placebo groups. Similarly, Lebowski et al. [5] reported no significant difference in 30-day postoperative mortality between the sugammadex and neostigmine groups.

The administration of sugammadex can reduce the incidence of RNB, which benefits patient recovery. However, perioperative outcomes result from the interaction of multiple factors. Age, frailty, and ASA classification are interrelated variables that collectively and significantly influence patient prognosis, particularly under surgical or physiological stress [50, 51]. Advancing age diminishes physiological reserves, thereby reducing tolerance, recovery capacity, and immune function. While these factors are often linked—where age predisposes individuals to frailty and a higher ASA class—each independently contributes to patient outcomes. Frailty denotes a state of heightened vulnerability due to declines in the physiological reserves of multiple systems, making individuals more susceptible to adverse health outcomes, even in response to minor stressors such as minor surgery or mild infection [52–55]. Furthermore, ASA classification has a strong positive correlation with complication and mortality rates [56]. Importantly, the co-occurrence of these factors exponentially increases risk rather than merely adding to it. Consequently, prognosis is based on this multifactorial framework. These factors may account for the significant heterogeneity observed in the meta-analysis results. Our study primarily included patients with ASA class 1–2, which may have contributed to the lack of variation in recovery outcomes due to the patients’ superior preoperative physical tolerance. Although the rapid reversal of neuromuscular blockade is an important component of enhanced recovery, it does not play a decisive role in overall patient recovery.

The impact of neuromuscular blockade antagonists on perioperative neurocognitive function in patients remains a subject of debate [45–47]. Previous studies suggest that cholinesterase inhibitors may reduce the incidence of POCD and POD [46, 48]. Recent evidence indicates that neostigmine influences immune-inflammatory activity through the cholinergic anti-inflammatory pathway (CAP), which may have implications for perioperative neurocognitive outcomes [57–61]. However, the exact mechanisms by which CAP mediates these effects are not fully understood. Although preclinical studies have demonstrated the compound’s dual role in regulating inflammation and providing cognitive protection in surgical contexts, significant translational gaps remain [62]. Importantly, existing evidence is largely derived from animal models, leaving unresolved issues regarding optimal dosing strategies, patient-specific responses, and long-term neurological effects in human populations. Our systematic review found no significant cognitive protective advantage of sugammadex compared to neostigmine or neostigmine compared to placebo. The studies included in our review primarily consist of single-center RCTs or extensive retrospective analyses, which often lack multi-center validation. Furthermore, the studied population is predominantly composed of patients undergoing surgery for specific conditions, limiting the generalizability of findings to the broader population [48]. Variability in the diagnostic scales used for cognitive assessment and the timing of evaluations across studies may also contribute to the heterogeneity of results.

Sugammadex incurs significantly higher per-dose costs compared to neostigmine, with price differences ranging from 20 to 30 times in some healthcare systems, thereby directly increasing perioperative expenses. Previous studies in bariatric surgery [63], hospital cost analyses [64], and single-center cost-effectiveness evaluations [65] confirm this economic disadvantage. However, sugammadex’s faster recovery profile may partially mitigate these costs in specific populations. These findings highlight two key imperatives: (1) sugammadex is a pharmacologically advanced option for high-risk patients for whom rapid recovery is essential, and (2) comprehensive cost-effectiveness analyses—especially those examining long-term recovery outcomes and context-specific value—are urgently needed to optimize its strategic implementation.

The high heterogeneity observed in our primary outcomes represents a significant limitation of this study, necessitating careful interpretation of the results. This variability suggests that the effectiveness of the intervention, such as different neuromuscular reversal strategies, may differ based on patient characteristics, surgical types, or specific anesthesia practices; therefore, our pooled effect estimates should be regarded as average effects. As a result, individualized clinical decision-making is essential, and the findings should not be universally applied to all patient populations. Nonetheless, the consistency of the primary effect across most subgroups reinforces the robustness of our main conclusions.

## Conclusion

Sugammadex demonstrated superior efficacy in reversing neuromuscular blockade compared to neostigmine, with a notable reduction in postoperative complications. However, this faster reversal did not translate into measurable improvements in broader recovery outcomes, such as hospital length of stay or overall recovery efficiency. Moreover, neither sugammadex nor neostigmine has been shown to significantly affect postoperative cognitive function, and neostigmine was not associated with improved cognitive outcomes. When economic considerations are set aside, sugammadex appears to offer a safer and more effective pathway for patient recovery than neostigmine, owing to its rapid and complete reversal of neuromuscular blockade. These findings highlight the clinical advantages of sugammadex while underscoring the need for further research to evaluate its cost-effectiveness and its potential influence on long-term recovery outcomes.

## Supplemental data

Supplemental data are available at the following link: https://www.bjbms.org/ojs/index.php/bjbms/article/view/12689/3972.

## Data Availability

The data associated with the paper are not publicly available; however, these are available from the corresponding author upon reasonable request.
